# P-1883. Evaluation of IVEnsure Device and Care Platform for Remote Therapeutic Monitoring in OPAT: Interim Analysis of a Pilot Study

**DOI:** 10.1093/ofid/ofae631.2044

**Published:** 2025-01-29

**Authors:** Mitchell Berenson, Jacob K Dozier, Emily A Siegrist, Justin D Dvorak, Debbie N Meyer, Bushra Siddique, Dale W Bratzler, Joseph Sassine

**Affiliations:** IVEnsure, Inc., Dallas, Texas; IVEnsure, Inc., Dallas, Texas; OU Health, Oklahoma City, Oklahoma; University of Oklahoma Health Sciences Center, Oklahoma City, Oklahoma; IVEnsure, Inc., Dallas, Texas; OU Health, Edmond Medical Center, Edmond, Oklahoma; Oklahoma University Health Sciences Center, Oklahoma City, OK; University of Oklahoma Health Sciences Center, Oklahoma City, Oklahoma

## Abstract

**Background:**

Data on medication non-adherence in outpatient parenteral antimicrobial therapy (OPAT) and the implications on clinical outcomes are lacking. Remote therapeutic monitoring (RTM) might offer additional insights into OPAT adherence. We present here a pre-planned interim analysis of a pilot study of IVEnsure, an RTM device, in patients receiving OPAT.

**Figure:**
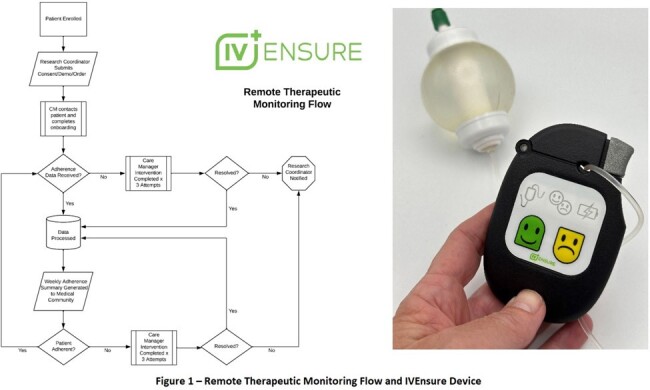

**Methods:**

This prospective study is recruiting adult patients discharged from a tertiary care center on OPAT starting July 2023. After consent, patients are enrolled in an RTM program, including the use of the IVEnsure device, and follow up with a case management team (Figure 1). This FDA-exempt and Medicare-approved healthcare remote monitoring device attaches to the tubing set of any IV medication and transmits infusion-related data in real-time via cellular transmission to generate weekly adherence reports. The study aims to analyze the effect of RTM on hospital readmission outcomes in patients on OPAT, compared to contemporaneous controls in an intention-to-treat analysis.

**Figure:**
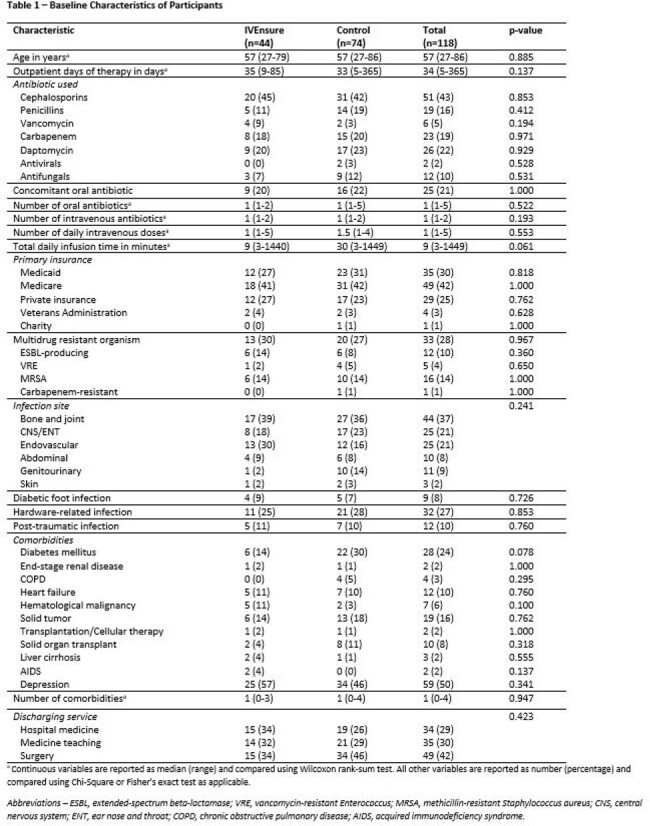

**Results:**

The interim analysis includes 44 patients (out of 90 planned) and 74 controls. Baseline characteristics are overall similar between both groups (Table 1). Adjusted for patient age at discharge, insurance status, infection class, presence of diabetes, and discharging service, RTM with IVEnsure is associated with a significant decrease in 90-day readmission rate related to the original infection or OPAT (4% vs. 23%, adjusted OR 0.14, 95% CI 0.02-0.54, p=0.01, Tables 2-3), leading to estimated cost savings of $213,825 in the IVEnsure arm, or $4,860 per patient (Table 4). Within the IVEnsure group, 45% of the patients required an intervention by the study team, 75% of which occurred in the first week of OPAT, resulting in improved or sustained adherence in 73% of the patients (Table 5).

**Figure:**
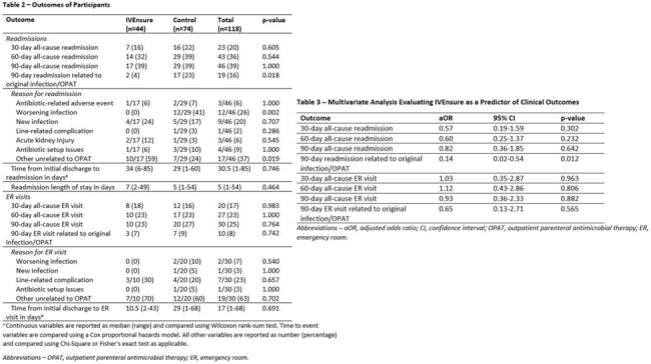

**Conclusion:**

RTM with IVEnsure significantly reduced infection-related readmissions and associated costs in patients on OPAT. Study accrual continues to evaluate additional readmission-related outcomes.

**Figure:**
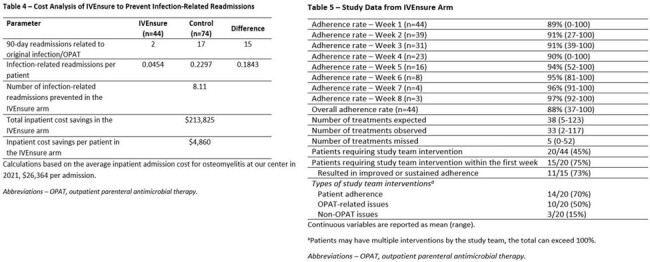

**Disclosures:**

Mitchell Berenson, MPH, Community Infusion Solutions: CEO|IVEnsure, Inc.: CEO Jacob K. Dozier, BS, Community Infusion Solutions: COO|IVEnsure, Inc.: COO Debbie N. Meyer, MS, IVEnsure, Inc.: Chief Technology Officer Joseph Sassine, MD, Ansun BioPharma: Grant/Research Support|Cidara Therapeutics: Grant/Research Support|Community Infusion Solutions: Grant/Research Support|F2G: Grant/Research Support|Shionogi: Grant/Research Support

